# A mapping-knowledge-domain analysis of ERP research on language processing

**DOI:** 10.3389/fnhum.2024.1352753

**Published:** 2024-06-12

**Authors:** Yi Sun, Xiaoyang Luo

**Affiliations:** Center for Linguistics and Applied Linguistics, Guangdong University of Foreign Studies, Guangzhou, Guangdong, China

**Keywords:** ERPs, language, systematic review, Citespace, bibliometrics

## Abstract

The event-related potentials (ERPs) technique represents a newly developed methodology in cognitive neuroscience and has significantly extended the scope of linguistic studies, offering valuable insights into cognitive processes related to language. While extant literature reviews have addressed specific facets of ERP research on language processing, a comprehensive overview of this domain remains notably absent. This study aims to fill this gap by pioneering a mapping-knowledge-domain analysis of ERP research on language processing using Citespace, a visualized bibliometric software. The current study conducted a meticulous survey and evaluation of relevant literature extracted from the Web of Science core collection. Initially, this study outlines the spatial-temporal distribution within this domain. Subsequently, employing document co-citation analysis, keyword co-occurrence analysis, cluster analysis, and burst detection analysis, this study delved deeper into the research landscape. Findings reveal that key areas in ERP research on language processing predominantly focus on sentence comprehension, reading comprehension, and mismatch negativity, with notable emphasis on topics such as speech perception, temporal dynamics, and working memory. The current study advocates for future investigations to concentrate on larger linguistic units, explore the integration of ERP components and their functional significance, and scrutinize individual differences among participants. These directions are imperative for advancing the understanding of language processing mechanisms.

## 1 Introduction

The human brain continuously generates neural activities, eliciting electrical signals that can be recorded using scalp electrodes. These signals correspond to individuals’ mental states and manifest as brain-wave patterns, recorded by electroencephalograms (EEGs). EEG plots offer information regarding amplitude, wavelength, and frequency of electrical signals. However, an excess of background information in EEGs hinders accuracy, limiting the detailed analysis of the brain’s response to specific stimuli. To address this limitation, researchers have turned to event-related potentials (ERPs) as a solution. ERPs encompass the brain’s electrical activity linked to specific sensory, cognitive, and motor events (Luck, 2014: 4). ERPs are obtained by recording EEG signals within defined time windows corresponding to stimuli. Typically, ERPs involve averaging across multiple trials to minimize the impact of extraneous factors and accentuate the primary effects of stimulus events (Gonzalez-Marquez et al., 2007: 405). Components of ERPs are denoted based on their polarities (negative or positive) and latencies (the time at which they peak after stimulus onset). For instance, the term “P300” designates a positive component occurring 300 ms after stimulus onset.

Modern research on ERPs commenced in 1964 when Gray Walter and his research team identified the first cognitive ERP component, termed the Contingent Negative Variation (CNV) (Walter et al., 1964). Since then, various ERP components have been extensively studied within the framework of language processing, including both written language comprehension and oral speech perception.

Among these, the N400 stands as a seminal contribution in linguistic research. Discovered in 1980 by Kutas and Hillyard, the N400 has emerged as one of the most crucial ERP components associated with language processing. It derives its nomenclature from its occurrence between 200 and 300 ms following stimulus onset, peaking around 400 ms. Kutas and Hillyard’s (1980) study initially linked this component to semantic violation. Over time, the N400 has been widely acknowledged as a measure of semantic processing (Kutas and Federmeier, 2011; Beres, 2017). Recent investigations have further elucidated the effects of the N400, encompassing auditory materials and extending to non-linguistic stimuli (Kutas et al., 1987; Strijkers and Costa, 2011).

Another significant ERP component, the P600, is intensively associated with syntactic processing. Initially detected in syntactically unacceptable or unpredictable sentences (Osterhout and Holcomb, 1992; Hagoort et al., 1993), the P600 subsequently manifested in sentences with intricate structures (Kaan et al., 2000). Thus, it can be inferred that the P600 primarily gauges the re-analysis of grammar or syntax.

The Mismatch Negativity (MMN) is a negative peak triggered approximately 100–250 ms after stimulus onset. It is widely acknowledged for its role in spoken language processing. The amplitude of MMN reflects the memory trace of language components in the human brain (Pulvermüller and Shtyrov, 2006). Expected stimuli typically evoke a less pronounced MMN compared to unexpected ones, with pseudo-words evoking a larger MMN than words (Chen et al., 2020).

The ERP technique has been extensively utilized across various dimensions of language processing research. Primarily, it serves as a valuable instrument for exploring the perception or comprehension of linguistic structures at multiple levels, encompassing phonetic (Sucevic et al., 2015; Whitten et al., 2020), lexical (Zhao et al., 2017; Xia and Peng, 2022), syntactic (Mascelloni et al., 2019; Deng and Zhu, 2020), and even discourse analysis (Li et al., 2018; Rasenberg et al., 2020). Furthermore, ERPs contribute significantly to interdisciplinary investigations within the realm of language processing. Researchers have directed their focus toward unraveling the neural mechanisms associated with bilingualism (Strijkers et al., 2010; Misra et al., 2012), emotional processing (Hinojosa et al., 2010; Chwilla et al., 2011), and language impairment (Leppanen et al., 2010; Olichney et al., 2011), among other domains.

Despite the substantial body of empirical studies, conducting comprehensive literature reviews remains important in elucidating the essence and trends within the subject matter. Existing literature reviews have delineated specific aspects of ERP research on language processing. Some have delved into the effects and functionalities of particular ERP components (Kaan et al., 2000; Pulvermüller and Shtyrov, 2006; Kutas and Federmeier, 2011), while others have assessed specific domains within ERP studies in linguistics (Morgan et al., 2020; Silkes and Anjum, 2021). However, a limited number of reviews offer a comprehensive overview of this research (Beres, 2017). Additionally, prior reviews mainly relied on researchers’ intuitions (Vigliocco et al., 2011), which might constrain the inclusion of all pertinent studies on the subject. Hence, the present study undertakes a systematic literature review of ERP research on language processing, employing a quantitative mapping knowledge domain methodology to address this gap.

Systematic reviews play a vital role in amalgamating findings from original studies within a specific research domain. These reviews encapsulate the consensus and knowledge gaps within the field, facilitating an evaluation of its trajectory and future prospects (Chen and Song, 2019). In comparison with conventional literature reviews, systematic reviews adopt a more stringent methodology, thereby yielding more substantial outcomes. Bibliometrics, as a quantitative tool, facilitates a comprehensive analysis of academic literature within a particular research sphere and conducts statistical analyses utilizing appropriate algorithms (Yuan and Sun, 2023). Consequently, mapping-knowledge-domain analysis serves as a means to graphically depict these findings. These visual representations illuminate various facets of the research field, including collaborative networks among scholars, social networks established through co-authorship, citation patterns, and co-citation networks (Chen, 2004). In essence, the process of mapping-knowledge-domain analysis enables the evaluation of extensive literature, particularly in research fields with broad coverage. Presently, mapping-knowledge-domain analysis has found widespread application in elucidating the developmental patterns and trends within various disciplines. For instance, in the realm of linguistics, Ahmed et al. (2022) demonstrated the developmental trajectories and emerging trends within cognitive linguistics.

Few studies were published in 1980s when ERP technique was first applied in linguistic research. However, such studies met with a significant increase as the technique became widely-used, especially after 2000. Therefore, the present study employs bibliometric methods to complete a mapping-knowledge-domain analysis, conducting a systematic review of ERP research on language processing in the past two decades (2002–2022). This investigation aims to scrutinize the developmental trajectory, current status, and potential future directions within this research domain. Specifically, our inquiry revolves around several research questions: (1) What is the temporal-spatial distribution of scholarly works pertaining to ERP research on language processing? (2) Which references, journals, and authors receive the highest citation frequencies within the research domain? (3) What are the prevailing focal areas and frequently debated topics within the field? (4) What prospective directions and potential trends characterize the landscape of ERP research on language processing?

## 2 Methods

### 2.1 Data collection

For the systematic review of ERP research on language processing, data were sourced from the Science Citation Index-Expanded (SCIE), the Social Science Citation Index (SSCI), and the Arts & Humanities Citation Index (A&HCI) within the Web of Science (WOS) Core Collection by Clarivate Analytics (2021). The Web of Science repository offers access to premier publications along with their citation data across the natural and social sciences, encompassing a time frame since 1975. Consequently, utilizing the core collection of this database ensures the inclusion of high-quality materials essential for this comprehensive analysis.

**FIGURE 1:**
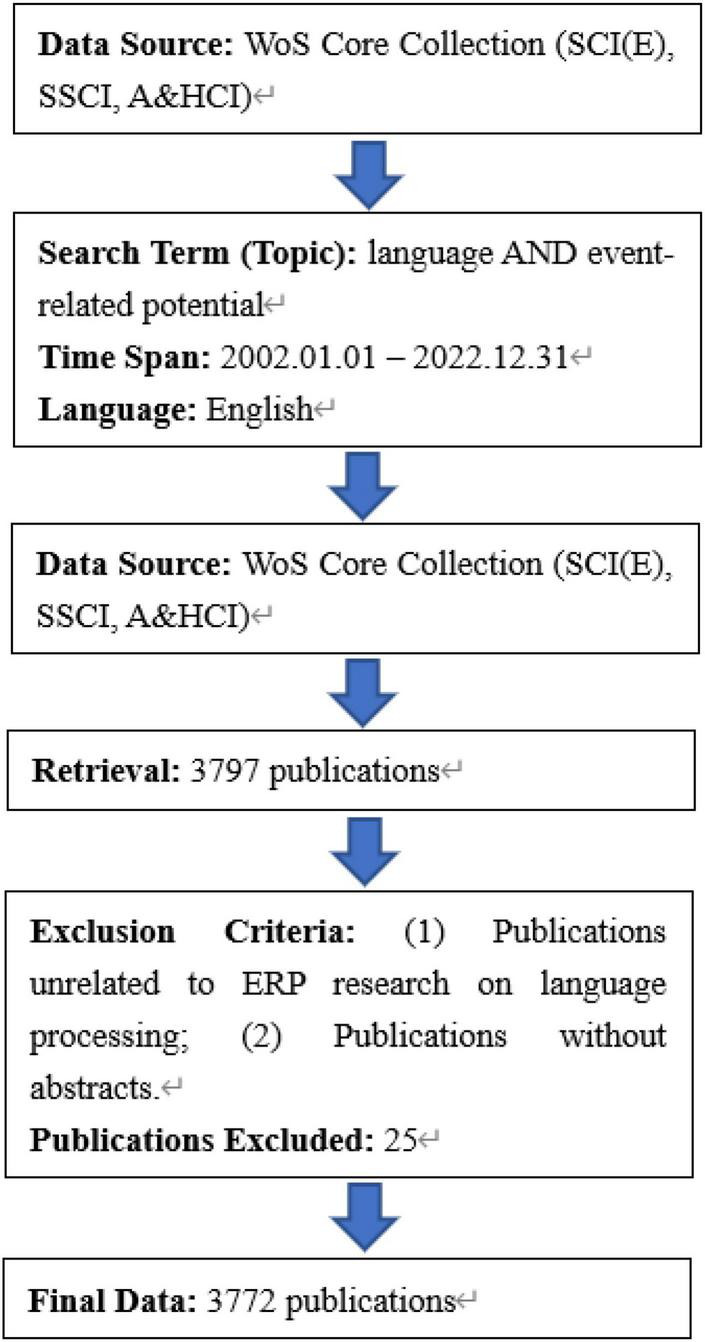
Procedure of data collection.

In accordance with the delineation and scope of the research domain, a systematic literature retrieval strategy was formulated as follows: Initially, keywords “language” and “event-related potential” were selected as primary retrieval terms, applied within the specified temporal boundary from January 1, 2002, to December 31, 2022. Subsequently, articles and reviews were exclusively considered in the document type, while the language criterion was restricted to English. 3797 publications were retrieved from the database. Following the online retrieval of literature, a meticulous manual screening process was executed based on predefined criteria to ensure the relevance and alignment with the subject matter. Two exclusion criteria are set to complete the screening: (1) Publications unrelated to ERP research on language processing. (2) Publications without abstracts. Records in either of abovementioned conditions were systematically excluded from the dataset. Consequently, a total of 3772 publications meeting the stringent inclusion criteria were identified for subsequent analysis. These selected publications were compiled and exported in a plain text file format, encompassing complete records along with cited references. The step-by-step protocol for data collection is presented in Figure 1.

### 2.2 Data analysis

In this study, a mapping-knowledge-domain analysis within ERP research on language processing was conducted employing Citespace 6.1.R6. Citespace is an information visualization software based on Java programming language. It enables the visualization of the knowledge structure and the identification of potential trends by employing co-citation analysis and pathfinder network scaling algorithms (Chen, 2016). Specifically, Citespace generates two distinct visual representations: one focused on co-citation analysis encompassing references, cited authors, and journals, while the other concentrates on co-occurrence analysis involving keywords, clusters, and citation bursts.

Preceding the formal analysis, duplicate records were eliminated using Citespace. According to the records in the current research, all 3772 publications were valid for the formal analysis. Then, several methods of mapping-knowledge-domain analysis were employed to address the pertinent research inquiries. To address questions (1), a comprehensive synthesis of the retrieved articles was presented, encompassing details such as geographic origins and publication timelines. For question (2), a co-citation analysis of references, cited authors, and journals was undertaken to identify the most frequently cited researchers and their contributions. To elucidate question (3), a co-occurrence analysis of keywords and clusters was executed to visually represent the primary themes encapsulated in the relevant studies. Finally, in addressing question (4), burst detection analysis of keywords was utilized to discern emerging trends across distinct temporal segments.

## 3 Results

### 3.1 The spatio-temporal distribution of publications

**FIGURE 2:**
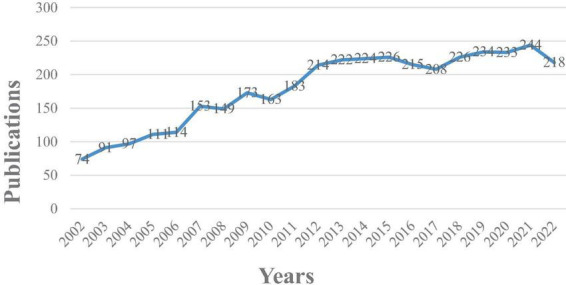
Temporal distribution of literature in ERP research on language processing.

To explore the temporal distribution of literature pertaining to ERP research on language processing, we analyzed the publication statistics across various years using data obtained from WoS. Figure 2 illustrates the chronological progression of ERP research on language processing from 2002 to 2022.

**FIGURE 3:**
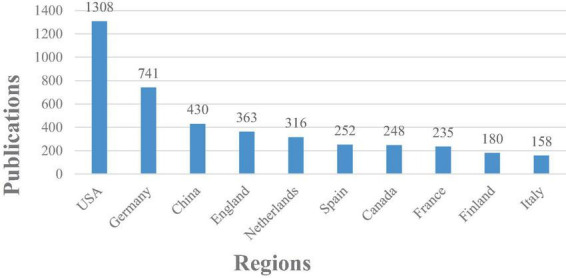
Regional distribution of literature in ERP research on language processing.

**FIGURE 4:**
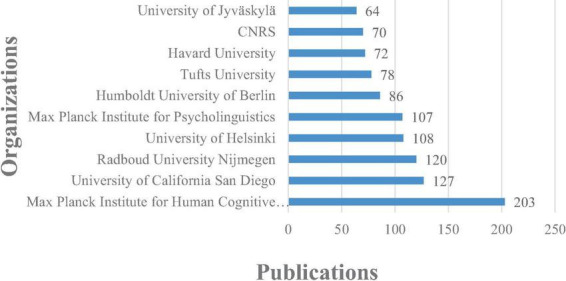
Productive organizations of literature in ERP research on language processing.

Figures 3, 4 illustrate the top 10 productive countries/regions and organizations between 2002 and 2022 in the research field. Through the examination of these statistics, a broad comprehension of the geographical distribution of ERP research on language processing can be obtained.

### 3.2 Co-citation analysis of references, cited authors and cited journals

**FIGURE 5:**
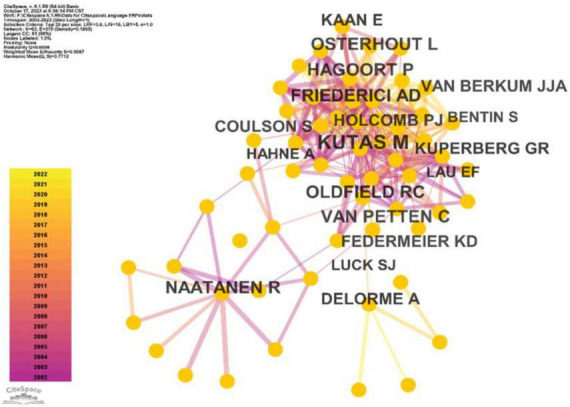
Frequently cited authors in ERP research on language processing.

**FIGURE 6:**
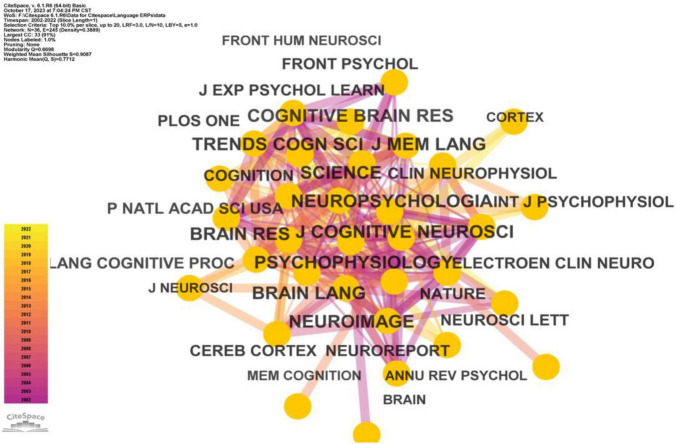
Frequently cited journals in ERP research on language processing.

**FIGURE 7:**
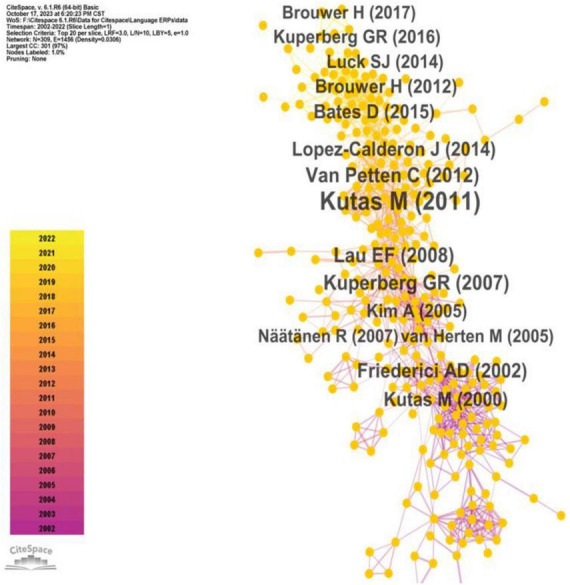
Frequently cited works in ERP research on language processing.

Co-citation analysis stands as a fundamental technique within bibliometric quantitative studies, designed to uncover the specialized domains within a scientific field (Morris and Van der Veer, 2008). This method often involves the identification of specialized clusters based on the co-citation of individual entities. For instance, Author Co-Citation Analysis (ACA) and Document Co-citation Analysis (DCA) are commonly utilized to delineate prominent researchers and their works, thus elucidating the knowledge network within specific research domains (Chen, 2006; Zhao and Strotmann, 2008; Chen et al., 2010). In this present study, we employed co-citation analysis to unveil the most frequently cited authors, references, and journals. Our analysis applied a 1-year time slice and selected the top 20 entities within each slice as parameters in Citespace. Figures 5–7 portray the outcomes of this analysis.

Figure 5 illustrates the leading authorities in ERP research on language processing. Based on the analysis conducted using Citespace, the top 10 scholars and their corresponding citation counts are as follows: Kutas (1832 citations), Friederici (1033 citations), Hagoort (958 citations), Oldfield (814 citations), Osterhout (785 citations), Näätänen (738 citations), Van Petten (700 citations), Holcomb (601 citations), Federmeier (597 citations), and Kuperberg (548 citations). These esteemed scholars have played instrumental roles in shaping the landscape of ERP research on language processing, contributing significantly to the foundational framework of this field.

Figure 6 delineates the most frequently cited journals in ERP research on language processing, signifying their concentrated focus and high caliber publications within this discipline. According to the statistical data derived from Citespace spanning the period from 2002 to 2022, the top 10 journals and their corresponding citation counts are as follows: Journal of Cognitive Neuroscience (3019 citations), Psychophysiology (2726 citations), Brain and Language (2650 citations), Neuropsychologia (2587 citations), Neuroimage (2179 citations), Science (2061 citations), Trends in Cognitive Sciences (2036 citations), Brain Research (2007 citations), Cognitive Brain Research (1886 citations), and Journal of Memory and Language (1872 citations).

Figure 7 visualizes the most referenced contributions within the domain of ERP research on language processing. These findings align with the outcomes obtained through co-citation analyses of referenced authors and journals. Eminent neurolinguistic scholars such as Friederici, Kutas, and Van Petten are prominently featured in these significant works. Additionally, these works are predominantly disseminated through influential journals like Trends in Cognitive Sciences, Brain Research, Science, among others.

### 3.3 Co-occurrence analysis of keywords

**FIGURE 8:**
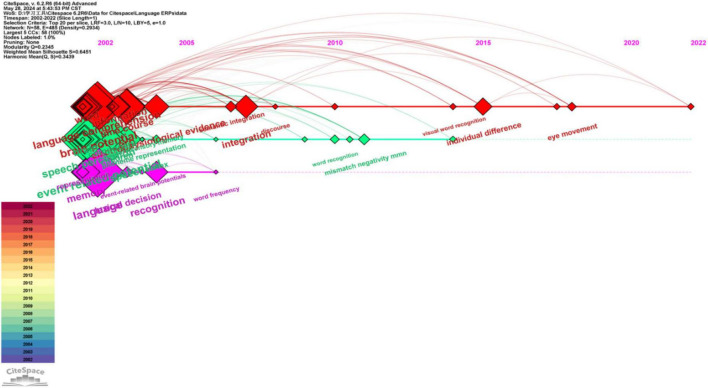
Diachronic view of the clusters in ERP research on language processing.

Keywords hold an essential role in demonstrating the primary theme and scope of a study. When two keywords frequently co-occur, it suggests a potential connection between them. It is commonly understood that the frequency of co-occurrence reflects the strength of their association. Within Citespace, the Betweenness Centrality stands as a metric to gauge this association strength. A higher centrality value assigned to a keyword signifies its relative significance within the network (Chen, 2004; Han et al., 2022; Peng and Hu, 2022). Therefore, this investigation undertakes a co-occurrence analysis to explore the centrality and frequency patterns of keywords in the domain of ERP research on language processing, aiming to identify prevalent focal points. Figure 8 visually presents the highlighted keywords and their interconnections, while Table 1 delineates the top terms alongside their respective frequencies and centrality values.

**TABLE 1:** Top keywords in ERP research on language processing.

Frequency	Centrality	Keywords	Frequency	Centrality	Keywords
2013	0.48	Event related potential	266	0.03	Memory
1049	0.11	Language	223	0.02	Electrophysiological evidence
841	0.12	Language comprehension	216	0.02	Integration
649	0.11	Speech perception	213	0.03	Activation
498	0.02	Time course	207	0.01	Information
360	0.06	Brain	195	0.01	Sentence comprehension
321	0.04	Word	192	0.01	Children
316	0.05	Working memory	183	0.04	Recognition
289	0.04	Mismatch negativity	157	0.03	Attention
286	0.03	N400	144	0.01	Individual difference
279	0.07	Speech	110	0.01	Response

**FIGURE 9:**
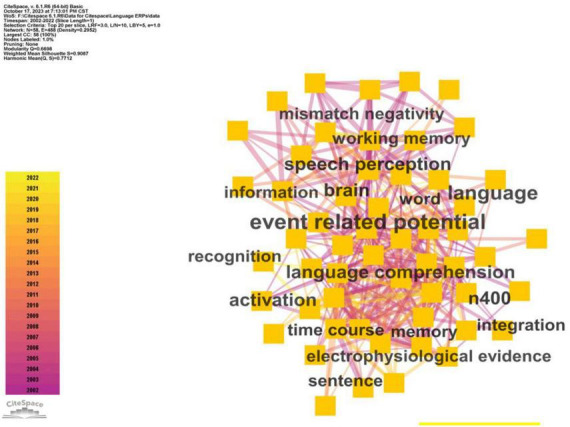
Keywords’ co-occurrence network in ERP research on language processing.

Referring to Figure 9 and Table 1, the predominant keywords in ERP research on language processing encompass event-related potential, language, language comprehension, speech perception, time course, brain, word, working memory, mismatch negativity, and N400.

### 3.4 Cluster interpretations

**FIGURE 10:**
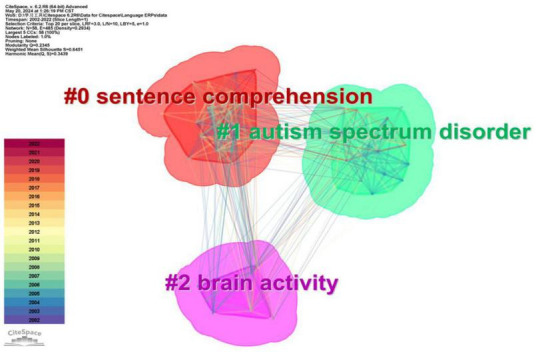
Synchronic view of the clusters in ERP research on language processing.

Based on the keyword co-occurrence analysis, this study performed a cluster analysis to categorize the terms derived from all 3772 publications. LLR (Log Likelihood Ratio) is a text analysis algorithm used in tasks like classification. It measures the likelihood of a term occurring in one class compared to others. High LLR values indicate terms with strong discriminatory power, aiding in feature selection for accurate classification. The generated clusters, labeled using LLR, resulted in three distinct clusters. Figures 8, 10 present both synchronic and diachronic perspectives of these clusters, while Table 2 encapsulates essential information regarding each cluster.

**TABLE 2:** Summary of clusters of keywords in ERP research on language processing.

Cluster ID	Size	Silhouette	Cluster label (LLR)	Top terms (LSI)	Top terms (LLR, p-level)
#0	26	0.658	Sentence comprehension	ERP study; event-related potential, ERP evidence; sentence comprehension; electrophysiological evidence; electrophysiological correlate; brain potential; second language; language processing; autism spectrum disorder	Sentence comprehension (7041.85, 1.0E−4); mismatch negativity (6590.45, 1.0E−4); syntactic processing (5019.05, 1.0E−4); ERP investigation (4944.53, 1.0E−4); autism spectrum disorder (4468.93, 1.0E−4)
#1	24	0.712	Autism spectrum disorder	Mismatch negativity; ERP study; autism spectrum disorder; event-related potential; electrophysiological evidence; sentence processing; late second language learner; ERP investigation; age-related change; absolute pitch	Mismatch negativity (10431.46, 1.0E−4); autism spectrum disorder (8214.98, 1.0E−4); syntactic processing (5842.38, 1.0E−4); ERP investigation (5261.58, 1.0E−4); auditory processing (4955.18, 1.0E−4)
#2	8	0.654	Brain activity	ERP study; event-related potential; event-related brain potential; event-related potential study; ERP evidence; event-related brain; potential study; semantic prediction; autism spectrum disorder; neural responses	Reading comprehension (2647.79, 1.0E−4); autism spectrum disorder (2010.04, 1.0E−4); semantic prediction (2008.43, 1.0E−4); predictability effect (1952.33, 1.0E−4); novel metaphor (1907.48, 1.0E−4)

### 3.5 Burst detection analysis of keywords

Over the past two decades, ERP research on language processing has witnessed significant development, marked by the publication of numerous studies. Our analysis using Citespace revealed that sentence comprehension, mismatch negativity, and reading comprehension have surfaced as focal points. In particular, discussion surrounding speech perception, working memory, and temporal dynamics has been prevalent. This field appears interdisciplinary, amalgamating theories and methodologies from diverse subjects. Hence, it can be inferred that ERP research on language processing presently holds a pioneering role.

**FIGURE 11:**
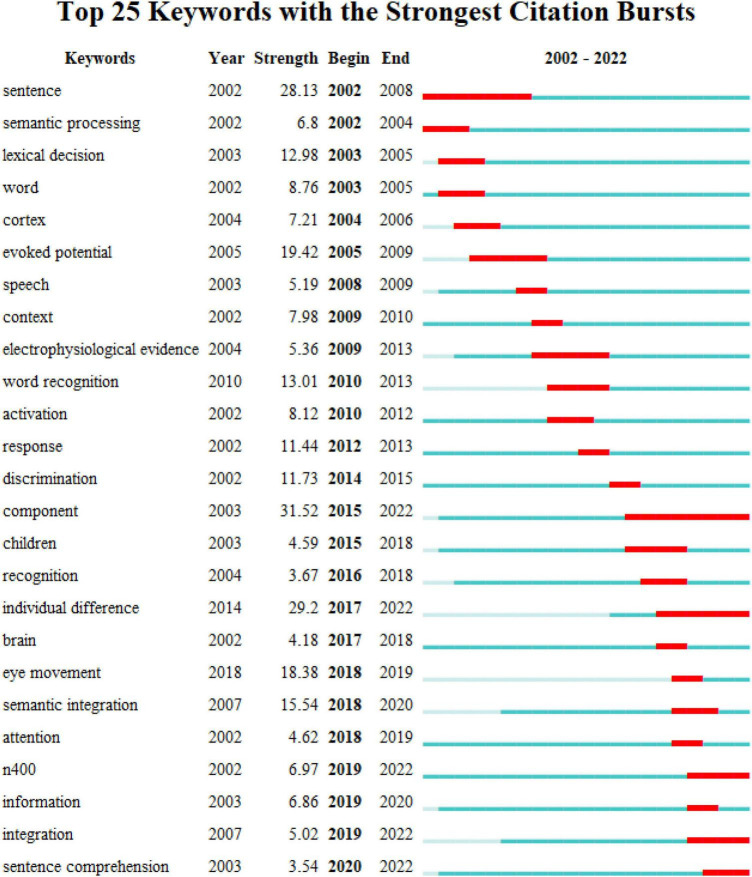
Burst terms in ERP research on language processing.

To delve deeper into the emerging trends within ERP research on language processing, our study conducted burst detection analysis on 3772 publications’ keywords. Burst terms represent keywords that undergo a remarkable surge within a specific timeframe, indicating ongoing developmental trends (Yuan and Sun, 2023). Figure 11 illustrates the top 25 burst terms:

Figure 11 presents the evolving trends in ERP research on language processing over the preceding two decades through the examination of burst terms observed in distinct time periods. In this figure, “year” represents the emerging time of the burst term, shown by deep blue column. “Begin” and “end” in red column demonstrates the burst period of the term. “Strength” refers to a measure that quantifies the intensity and significance of the sudden increase in frequency of the term over its burst period. Citespace utilizes an algorithm developed by Jon Kleinberg for burst detection (Kleinberg, 2002).

## 4 Discussion

### 4.1 The spatial-temporal distribution of ERP research on language processing

The spatial-temporal distribution of the research pattern is clearly revealed by the visualization data. Figure 2 depicts a consistent upward trend in international publications focusing on ERP research on language processing. Initially emerging as a novel methodology, there existed a relatively limited number of studies employing ERPs in language processing research. Notably, between 2002 and 2004, the yearly publication count remained below 100. However, the field witnessed a subsequent surge in research endeavors. Particularly noteworthy are significant increments observed during three distinct periods: 2006 to 2007, 2010 to 2012, and 2017 to 2021. Remarkably, in 2021, the publication count reached its zenith, registering 244 publications within a single year. Despite minor declines in publication counts observed in 2008, 2010, and 2017, each downturn was promptly followed by obvious upswings. These observations underscore the emergence and burgeoning interest in ERP research on language processing in recent years. Notably, it is posited that significant discoveries concerning ERP components and advancements in research methodologies have propelled the proliferation of studies in this field (Kutas and Federmeier, 2011; Lopez-Calderon and Luck, 2014; Bates et al., 2015). Consequently, the number of publications experiences substantial growth during specific temporal periods.

From the insights gleaned in Figures 3, 4, a dominant presence is observed in ERP research on language processing, particularly attributed to the United States. Over a span of two decades, US-based researchers have contributed significantly, accounting for approximately 35% of the total studies in this field, amassing a total of 1308 publications. Outstanding academic institutions such as the Max Planck Institute for Human Cognitive and Brain Sciences (with 203 articles), the University of California San Diego (with 127 articles), and the Radboud University Nijmegen (with 120 articles) have significantly bolstered the advancement of ERP research on language processing. Geographically, the epicenters of ERP research on language processing are chiefly concentrated in Europe and North America. Nevertheless, an encouraging trend is observed in Asia, particularly in China (with 430 articles), evidencing substantial growth and promising advancements in catching up with the forefront of ERP research.

### 4.2 Research focuses on ERP research on language processing

Results from co-citation analysis of references (Figure 7 and Table 3), co-occurrence analysis of keywords (Figure 9 and Table 1) and cluster interpretation (Figures 8, 10 and Table 2) have unraveled several key points in the ERP research on language processing from 2002 to 2022. Discussion will concern three main topics, namely the ERP components, methodologies and techniques, as well as research scope.

**TABLE 3:** Top 10 cited works in ERP research on language processing.

Citation count	Author (year)	Publication name	Journal or press
300	Kutas and Federmeier, 2011	Thirty years and counting: Finding meaning in the N400 component of the event-related brain potential (ERP)	*The Annual Review of Psychology*
97	Van Petten and Luka, 2012	Prediction during language comprehension: Benefits, costs, and ERP components	*International Journal of Psychophysiology*
94	Lau et al., 2008	A cortical network for semantics: (de)constructing the N400	*Nature Reviews Neuroscience*
92	Kuperberg, 2007	Neural mechanisms of language comprehension: Challenges to syntax	*Brain Research*
83	Friederici, 2002	Toward a neural basis of auditory sentence processing	*Trends in Cognitive Science*
79	Lopez-Calderon and Luck, 2014	ERPLAB: an open-source toolbox for the analysis of event-related potentials	*Frontiers in Human Neuroscience*
77	Bates et al., 2015	Fitting linear mixed-effects models using lme4	*Journal of Statistical Software*
76	Kutas and Federmeier, 2000	Electrophysiology reveals semantic memory use in language comprehension	*Trends in Cognitive Sciences*
67	Brouwer et al., 2012	Getting real about semantic illusions: Rethinking the functional role of the P600 in language comprehension	*Brain Research*
61	Luck, 2014	An Introduction to the Event-Related Potential Technique (second edition)	The MIT Press

Several ERP components have been instrumental in exploring the neurocognitive mechanisms of language processing. The N400, illustrated by Kutas and Federmeier (2000) as an index of semantic processing, is a significant electrophysiological signature in language processing. Studies highlight its role in sentence processing and neural mechanisms of language comprehension, suggesting a dynamic and modality-specific semantic processing network predominantly in the left hemisphere (Koelsch et al., 2004; Frank et al., 2015). Kutas and Federmeier (2011) extended this by demonstrating that the N400 is evoked by multimodal stimuli, including written, spoken, and sign languages, even non-linguistic stimuli like drawings and videos. This broadened the exploration of the N400 into perception, attention, and memory, reflecting how the brain utilizes top-down and bottom-up information to understand the world. Lau et al. (2008) anatomically deconstructed the N400 component, proposing a neurocognitive model for semantic processing that includes core regions like the left posterior temporal cortex (lPTC), left anterior temporal cortex (lATC), angular gyrus (AG), and left inferior frontal gyrus (lIFG). This model suggests that lexical semantic information is stored in the lPTG and higher-level semantic processes involve the lATG and AG, with the lIFG implicated in selecting and retrieving specific lexical representations.

The P600, another crucial ERP component, normally associated with syntactic processing, was interpreted by Brouwer et al. (2012) through the Retrieval-Integration Account. They found that semantically anomalous sentences evoked a P600 rather than the expected N400, suggesting that the P600 reflects the integration of lexical information within contexts and plays a role in constructing or updating mental representations.

Mismatch negativity (MMN) is closely related to sound processing and signifies the activation of memory networks for language sounds and spoken words (Pulvermüller and Shtyrov, 2006). Shtyrov and Pulvermüller (2002) observed distinct MMN responses to word stimuli compared to pseudowords, supporting immediate word processing upon identification. Jacobsen et al. (2004) demonstrated that MMN is evoked by low-frequency vowels, indicating that the auditory processing network can pre-attentively extract vowel formant structures. MMN also indicates syntactic processing. Hahne et al. (2001) found that combined physical and syntactic violations evoked larger MMNs than either alone, suggesting parallel and independent early-stage processing. Pulvermüller et al. (2007) evidenced the autonomy of syntactic processing, while Hasting et al. (2007) revealed functionally distinct neural mechanisms underlying subject-verb agreement and word class information. What’s more, studies on MMN encompass multimodal materials (Chen et al., 2022). Ishida and Nittono (2022) explored anomalies in music, finding that syntactic anomalies evoke early right anterior negativity (ERAN) and acoustic anomalies trigger MMN. Ding et al. (2022) showed that neutral and fear emotions induce greater MMN compared to positive emotions, revealing a processing bias toward negative emotions in body gestures.

The Left Anterior Negativity (LAN) and Early Left Anterior Negativity (ELAN) are components associated with syntax comprehension, typically observed over the left anterior scalp regions. ELAN, appearing within 100–200 ms after stimulus onset, is involved in early syntactic processing, while LAN, occurring around 300–500 ms, reflects sensitivity to syntactic violations or grammatical errors (Palolahti et al., 2005; Steinhauer and Drury, 2012).

Methodologies in ERP research are crucial for study quality. Luck (2014) provides a comprehensive guide on ERPs in cognitive neuroscience, covering theoretical foundations, experimental paradigms, and data analysis techniques. Lopez-Calderon and Luck (2014) introduced ERPLAB, an open-source toolbox for ERP data analysis compatible with MATLAB, enhancing the ease of offline analysis of ERP data. Bates et al. (2015) detailed the application of linear mixed-effects models using the lme4 package in R, offering a more refined model for predicting underlying patterns compared to traditional ANOVA analysis.

ERP studies on language processing often use visual stimuli, emphasizing reading comprehension. Ditman et al. (2007) proposed the self-paced reading design in ERP research for a more precise examination of reading processing. Metzner et al. (2017) integrated ERPs and eye-movement techniques, suggesting alternative strategies in sentence comprehension, such as reanalysis (P600) or tolerance of insufficient interpretations (N400).

Novel methods like time-frequency analysis and microstate analysis are gaining attention. Time-frequency analysis decomposes ERPs into frequency components over time, offering insights into cognitive functions (Herrmann et al., 2014; Morales and Bowers, 2022). Microstate analysis studies dynamic brain activity associated with cognitive processes, segmenting the ERPs signal into temporally stable topographic states, representing synchronized neural activity (Ott and Jäncke, 2013; Croce et al., 2021).

The ERP technique has found diverse applications in various linguistic research domains. Van Petten and Luka (2012) synthesized ERP studies on prediction during language comprehension, noting the N400 as an indicator of benefits from semantic context and positive components like P300 and P600 addressing prediction costs. Friederici (2002) emphasized the neural underpinnings of auditory sentence processing, positing that comprehension is organized within a bilateral temporo-frontal network. Kuperberg (2007) reviewed studies on the P600, suggesting that language comprehension involves competing neural processing streams: a semantic stream reliant on long-term memory and a combinatorial stream for morphosyntactic rules. This clash triggers reanalysis, evident in the P600. Chow and Phillips (2013) investigated the “Semantic P600” phenomenon in Chinese, finding that well-formed role-reversed sentences evoked P600 activation, highlighting the dependence of online semantic processing on surface syntax. Deng et al. (2016) revealed early syntactic processing (ELAN) and semantic-syntactic integration (N400) in Chinese verbs. Stowe et al. (2018) emphasized the need for updated experimental designs to explain late components in sentence wrap-up. Recent studies highlight pragmatic knowledge’s role in sentence comprehension. Kuperberg et al. (2007) proposed an animacy-based P600 effect influencing thematic-semantic relations. Van Berkum et al. (2007) posited active referential processing during sentence comprehension. Casado et al. (2020) revealed that motor sequencing affects early syntactic processing. Figurative language studies by Ferretti et al. (2021) and Shen et al. (2022) focused on proverbs and metaphors in sentence processing. Studies on reading comprehension and development, such as Stites and Laszlo (2017), linked ERP components with children’s reading development, finding that N250 predicted phonological awareness. Tabullo et al. (2020) found that higher reading skills were associated with reduced N400 during expected word processing. Troyer et al. (2022) explored hemispheric processing asymmetries, highlighting the left hemisphere’s engagement with specific semantic information and the right hemisphere’s involvement in broader semantic connections.

### 4.3 Trends and prospects in ERP research on language processing

Referring to the outcomes from burst detection analysis (Figure 11), several findings of developing trends are discovered. Besides, future directions may be predicted based on the statistics.

The first finding denotes a transition toward examining larger linguistic units (Nieuwland et al., 2019). Throughout the early 21st Century, ERPs investigations in language predominantly centred on lexical processing, evident from burst terms like “lexical decision” (2003–2005) and “word” (2003–2005). Initial studies were primarily focused on unraveling the neurocognitive mechanisms involved in processing individual words, pivotal in understanding language structures. During this phase, some studies isolated word stimuli for examination (Barber et al., 2004; Carreiras et al., 2005), while others incorporated these stimuli into phrases or sentences (Allen et al., 2003; Weber-Fox et al., 2003; Schiller, 2006). Nonetheless, the primary emphasis remained on the lexical level. The proliferation of studies concentrating on lexical processing established a robust foundation for ERP research on language processing. Consequently, subsequent investigations broadened their scope to encompass varied linguistic materials, highlighted by the emergence of the burst term “sentence comprehension” (2020–2022). Beyond lexical-semantic attributes, sentence processing encompasses the integration of diverse linguistic information. Recent studies have frequently delved into neural mechanisms associated with syntactic processing (Jackson et al., 2020; Hao et al., 2021) and pragmatic processing (Allegretta et al., 2021; Jouen et al., 2021; Schoknecht et al., 2022).

The second key finding underscores the growing integration and synthesis observed in ERP research on language processing. Initially, most studies were centred around specific ERP components, aiming to explain their distinct functions. For instance, early investigations proposed the N400 as an indicator of semantic violation (Quiroz-G, 2003) and the P600 as a marker of syntactic violation (Kuperberg et al., 2003). This trend is evident from burst terms like “semantic processing” (2002–2004) and “evoked potential” (2005–2009). In more recent years, the emergence of new burst terms such as “component” (2015–2022), “N400” (2019–2022), and “integration” (2019–2022) has spurred a re-examination of ERP components. A substantial body of studies now argues that the same component may exhibit diverse effects or functions across different experimental contexts (Kutas and Federmeier, 2011; Niharika and Rao, 2020). Moreover, the appearance of the burst term “eye movement” (2018–2019) suggests a trend toward integrating the ERP technique with other methodologies in linguistic research to acquire more robust evidence (Kessler et al., 2021).

The third significant finding highlights the increasing attention given to individual participant characteristics in ERP research on language processing. Burst terms such as “individual difference” (2017–2022) and “children” (2015–2018) signify this emerging trend. Investigations into second language acquisition have begun to identify individual variations by considering between-subject factors such as second language ability (Bice and Kroll, 2021; Liang and Chen, 2022; Grey, 2023). Furthermore, discussions regarding individual factors like gender or age have gained traction in recent studies (Cheimariou et al., 2019; Choisdealbha et al., 2022; Padrón et al., 2023).

In general, ERP research on language processing is poised to advance along several key trajectories in the future. Firstly, there will be a continued trend toward investigating larger linguistic units, so as to find out human brain’s processing mechanisms of complex language structures. Secondly, there will be a synthesis and re-examination of ERP components, exploring their multifaceted nature and roles across different linguistic contexts. Thirdly, there will be an increased focus on individual participant characteristics, to better understand variability in language processing outcomes. Lastly, there will be a growing integration and update of ERP techniques with other methodologies, to provide a more comprehensive understanding of language processing mechanisms. Overall, these trends are expected to aid the development of more nuanced models of language processing.

### 4.4 Strengths and limitations

This study is the first mapping-knowledge-domain analysis on ERP research on language processing, which pioneers the systematic review of the topic. 3772 relevant publications from the WoS core collection are exclusively extracted for precise visualization analysis.

The database in the current study is still far from inclusive, with only WoS core collection included. Publications from other databases are not considered properly, for example, PubMed and Scopus. Besides, merely publications written in English have been selected, which might lead to a bias in the analysis. Finally, the research scope in the current study is limited in language comprehension and perception. However, in language-related ERP study, language production also plays an essential role. Further research should discuss the neurocognitive mechanisms of language production in detail.

## 5 Conclusion

Appearing from cognitive neuroscience, the Event-Related Potentials (ERPs) technique has emerged as an elite tool in linguistic research. Offering feasibility and high temporal precision, this technique provides a unique vantage point for probing the neural underpinnings of language processing. Despite a burgeoning number of studies in ERP research on language processing since the turn of the 21st century, there exists a dearth of systematic reviews applying bibliometric methods to offer a comprehensive overview of this thriving research domain. This study undertook a mapping-knowledge-domain analysis using Citespace, a visualization-centric bibliometric tool, to analyze 3772 relevant publications in the recent two decades (2002–2022) in the core collection of Web of Science. Several key findings have emerged from this comprehensive analysis. Over the past two decades, the volume of publications in ERP research on language processing has shown a consistent upward trajectory. Firstly, the United States and the Max Planck Institute for Human Cognitive and Brain Sciences were identified as the leading contributors in terms of publications in this field. Co-citation analysis illuminated the frequently cited authors, journals, and references, outlining the fundamental pillars of ERP research on language processing. Subsequent co-occurrence and cluster analyses of keywords highlighted pivotal topics and current focal points in this domain. Prominent keywords encompassed themes such as working memory, speech perception, and time course, among others. Three primary clusters surfaced: sentence comprehension, mismatch negativity, and reading comprehension. Moreover, a burst detection analysis underscored potential future directions, suggesting the ascension of larger linguistic units as a burgeoning interest. Beyond individual words and sentences, this trajectory endeavors to unravel the intricate neurocognitive processes underlying more extensive language structures such as paragraphs and discourses. Another critical area involves integrating ERP components and delineating their functional roles, thereby enriching our understanding of how these components collectively contribute to cognitive functions like syntactic and semantic integration. Furthermore, exploring individual differences within ERP profiles, encompassing factors such as linguistic proficiency and cognitive styles, promises to refine the applicability and relevance of research findings, potentially paving the way for personalized models of language processing.

The current study draws a picture of the existing status in ERP research on language processing, which may shed light on following research in the future. Although there are some limitations, this work tries to provide implication for future research in the field.

## Data availability statement

The original contributions presented in this study are included in the article/supplementary material, further inquiries can be directed to the corresponding author.

## Author contributions

YS: Conceptualization, Funding acquisition, Project administration, Resources, Supervision, Writing – review and editing. XL: Conceptualization, Data curation, Methodology, Writing – original draft, Writing – review and editing.
